# Antibacterial activity of plant-derived compounds and cream formulations against canine skin bacteria

**DOI:** 10.1007/s11259-024-10324-0

**Published:** 2024-02-07

**Authors:** Viola Strompfová, Lucia Štempelová, Tomáš Wolaschka

**Affiliations:** 1 Centre of Biosciences of the Slovak Academy of Sciences, Institute of Animal Physiology, Šoltésovej 4-6, Košice, 040 01 Slovakia; 2 Department of Pharmaceutical Technology, Pharmacognosy and Botany, University of Veterinary Medicine and Pharmacy, Komenského 73, Košice, 041 81 Slovakia

**Keywords:** Skin, Dog, Antibacterial agent, Cream, Plant compounds

## Abstract

An urgent need to find alternative antimicrobial compounds effective in the prevention and treatment of skin infections led us to study the inhibitory activity of eight plant-derived bioactive compounds (betulin, curcumin, glycyrrhizic acid, guaiazulene, piperine, quercetin, quinine, tannic acid) against 14 canine skin isolates (11 Gram-positive and three Gram-negative bacteria) selected based on antibiotic resistance and virulence features. The minimum inhibitory concentration (MIC) and the minimum bactericidal concentration (MBC) were determined using the broth microdilution method. In detail, the results for the eight different plant compounds showed their inhibitory activity in the concentration range from 0.04 to more than 16 mg/ml (MIC) and from 0.25 to more than 16 mg/ml (MBC). The most potent compounds appear to be tannic acid, followed by quinine and curcumin (MIC 0.04–16.0 mg/ml). The most susceptible strain to the tested agents in general was *Bacillus cereus* AE13, while *Enterococcus faecium* AA14 was the most resistant strain (the highest MICs) among the tested bacteria. The two most potent plant-derived compounds (tannic acid and quinine) were tested in mixture in different ratios (1:1, 1:2, 2:1). The lowest MIC and MBC values were observed for the 1:2 ratio, which was used for preparation of creams with different cream bases. One of the cream formulations (cream F) was effective up to 63.0 mg/ml (MIC) with a microbial inactivation time of 1–6 h according to the tested strain. This study provides evidence that some plant-derived compounds could have an antimicrobial effect against canine skin bacteria, the strength of which is bacterial strain dependent.

## Introduction

Contemporary lifestyle and urbanisation bring many potential risks to the health of concurrently bred pets and also have a significant impact on skin health. The extraordinary need for attention to skin health in dogs is evidenced by the very frequent occurrence at present of skin problems in dogs in the USA (Nationwide 2019) as well as in Europe (O’Neill et al. 2014). Skin allergies, ear infections and skin infections have the highest prevalence rates among them. The most common causes of these problems include parasites (fleas), environmental irritants (pollen, dust mites), food allergens and autoimmune reactions (Vetsnow, 2020). However, many skin symptoms also appear secondarily as a result of another primary disease. Similarly as in the case of the gastrointestinal tract, the commensal microbiota colonising the skin is also important for maintaining epithelial homeostasis and the overall health of the tissue (Coates et al. 2019). The symbiotic microorganisms occupying the skin niche prevent invasion by pathogenic or harmful organisms and are involved in the modulation of innate and adaptive immune responses (Grice and Segre 2011). Various endogenous (genetics, age, sex) or exogenous factors (cosmetics, antibiotics) may disrupt the host–microorganism relationship (Cusco et al. 2017). These shifts in microbial structure lead to dysbiosis, which can the lead to dermatological problems. Within the major bacterial strains dominating on the skin (Firmicutes, Proteobacteria, Fusobacteria, Actinobacteria and Bacteroidetes), the species distribution and abundance of Firmicutes, in particular of the family Staphylococcaceae and Streptococcaceae, is commonly altered during bacterial dysbiosis on the skin in dogs (Pierezan et al. 2016). The study of Chermprapai et al. (2019) analysing the skin microbiome using 16 S rRNA sequencing showed that *Staphylococcus*, *Psychrobacter*, *Trichococcus*, *Brachybacterium* and *Porphyromonas* were the most abundant bacteria in atopic skin in dogs. The same study also indicated the antimicrobial potential of malaseb shampoo use (miconazole and chlorhexidine) on the microbiota composition. Differences in species richness was also seen between healthy and allergic dogs, with allergic dogs having lower species richness in a further study (Hoffmann et al. 2014), which reported that allergic dogs had lower proportions of the Betaproteobacteria *Ralstonia* spp. when compared to healthy dogs. It is also necessary to mention the fact that there is a large inter-individual variability and differences among skin sites (Cuscó et al. 2017). The clinical significance of these dysbiotic changes, whether it is a cause or an effect, as well as many other important aspects remain unanswered. Nevertheless, skin microbiota has been shown to be an important clinical tool in the diagnosis and therapy of dermatological diseases and is a promising area for finding ways to prevent skin diseases.

Despite reductions in total antibiotic consumption in the European Union between 2011 and 2020, especially during the Coronavirus Disease 2019 (COVID-19), antibiotic resistance (AR) has increased for many antibiotic-bacterium combinations since 2011 (AR in the EU, EEA 2022). Of particular concern is the increase in resistance to the critically important antibiotics used to treat common infections related to health care. Although recent trends are encouraging, resistance to commonly used antibiotics remains high (> 20–50%) or very high (> 50–70%), and there are significant regional differences within the EU. AR is therefore still significant global health concern that affects both human and animal populations. It requires a collaborative One Health approach involving multiple participants, such as veterinarians, healthcare professionals, researchers and policymakers. As there is today a requirement to reduce the use of chemicals/antibiotics to the maximum possible extent, alternative antimicrobial compounds could be considered as replacements for them within control strategies. In this context, plant-derived bioactive compounds could represent a natural and safe way to manage skin infection without side effects. The World Health Organization (WHO) has stated that 80% of the developing world still benefits from the use of traditional medicines derived from medicinal plants. It recorded the names of over 20,000 species of medicinal plants and described them as potential sources of new drugs (Yadav and Agarwala 2011). Medicinal plants have enormous potential for the discovery of new bioactive compounds which can have antibacterial activity among other biological activities, such as anti-inflammatory, antioxidant, analgesic, etc. Plant-derived bioactive compounds of therapeutic value are mostly secondary plant metabolites that result from secondary plant metabolism and can occur as intermediate or end products. Although plant-derived chemicals have many different mechanisms of antimicrobial activity, the primary target site is the cytoplasmic membrane, affecting its structure, integrity, permeability or functionality (Savoia 2012). Since many of them have antimicrobial activity against multidrug resistant bacteria, most current research is focused on their possibility to replace antibiotics in this drug resistance crisis. The advantage of plant-derived compounds treatments is that they do not normally cause resistance (Lewis and Ausubel 2006).

The present study evaluated the efficacy of eight plant compounds against 14 canine skin bacteria showing pathogenic potential and multi-resistance. The most potent compounds were the basis for cream formulations tested for MIC and MBC values and inactivation time against selected bacteria.

## Materials and methods

### Bacterial strains and growth conditions

Fourteen different bacteria selected from a group of 98 isolates obtained from healthy canine skin and from a group of 20 strains obtained from skin lesions of dogs with dermatological diseases (atopic dermatitis and otitis externa) were used in antimicrobial assay. The selection was based on their antibiotic resistance and virulence factors. The following strains were used: *Bacillus cereus* AE13, *Enterococcus faecium* AA14, *Escherichia coli* EL13, *Klebsiella oxytoca* AX10, *Pseudomonas aeruginosa* BAS1, *Staphylococcus aureus* BO4, CE24, *S. capitis* ME17, *S. epidermidis* AL7, *S. haemolyticus* Ri3, *S. hominis* AG3, *S. pseudintermedius* OG9, TB13, *Streptococcus canis* BS10. These strains were collected in the period of one year (2020) in the eastern region of Slovakia (Štempelová et al. 2022). The identification of selected indicator bacteria was performed using Maldi-Tof MS on a Microflex LT instrument (Bruker Daltonic GmbH, Leipzig, Germany), and their phenotypic and genotypic characteristics (haemolytic activities, antibiotic resistance, production of slime, gelatinase, DNAse, caseinase, lipase, enzymes, presence of enterotoxin genes, biofilm formation) were described in our previous publication (Štempelová et al. 2023). Frozen cultures stored at -80 °C were revived by inoculation into Mueller-Hinton broth (MH broth, Bio-Rad Laboratories, USA) and incubation at 37 °C for 24 h.

### Preparation of tested solutions

For determination of the inhibitory activity of plant compounds (all from Sigma-Aldrich, USA), betulin (≥ 98%, B-9757-1G), curcumin from *Curcuma longa* (Turmeric, powder, C1386-10G), glycyrrhizic acid ammonium salt from glycyrrhiza root (liquorice, ≥ 95.0%, 50531-10G), guaiazulene (99%, G11004-10G), piperine (≥ 97%, P49007-5G), quercetin (≥ 95%, HPLC, solid, Q4951-10G), quinine (anhydrous, ≥ 98.0%, 22620-5G) and tannic acid (ACS reagent, 403040-50G) were used. The solutions were prepared according to plant compound solubility. The tannic acid was prepared in MH broth to a concentration of 16 mg/ml, while quinine was diluted 1:8 in ethanol (1:8 MH broth). All other compounds were diluted in dimethyl sulfoxide (1:8 MH broth) to a concentration 32 mg/ml.

### Determination of minimum inhibitory (MIC) and bactericidal (MBC) concentrations

The MIC and MBC were determined in triplicates according to the broth microdilution method (in sterile flat bottom 96-well microplates, Nunc, USA), with a final volume of 150 µl per well (Balouri et al. 2016, with minor modifications). The inoculum was prepared by dilution in MH broth to give a final organism density of approximately 5 × 10^5^ CFU/ml in the well (0.5 McFarland to have 1 × 10^8^ CFU/ml, then to dilute 1:100 with broth and then 1:1 to put 75 µl to well with 75 µl of broth with compound). The prepared solutions (150 µl) in the wells were diluted two-fold (transferring 75 µl). The following concentrations were tested: 16, 8, 4, 2, 1, 0.5, 0.25, 0,125, 0.06, 0.03 mg/ml. MH broth alone (growth medium without any bacteria and antimicrobial compound), the tested compound solution alone (growth medium with compound but without bacteria) and the medium inoculated with the tested strain (bacterial suspension without any antimicrobial compounds) were used as controls. Plates were incubated at 37 °C for 24 h. After incubation, the plates with plant compounds were coloured after the addition of 10 µl of resazurin (337.5 mg of resazurin powder in 50 ml of sterile distilled water, Serva, Germany) and incubated at 37 °C for 3 h for observation of the colour change. The MIC was determined as the lowest concentration that inhibited growth of the strain (a clear broth suspension or last a purple well prior to pink). The MBC was determined as the lowest extract concentration killing 99.9% of the bacterial inoculum after 24 h incubation at 37 °C according to the method of Mogana et al. (2020). The MIC and MBC values were expressed as the arithmetic means of the triplicate tests and the standard deviation. We evaluated antibacterial activity according to the MBC/MIC ratio. If the MBC/MIC ratio was ≤ 4, the effect was considered to be bactericidal, but if the MBC/MIC ratio was > 4, the effect was defined as bacteriostatic (Mogana et al. 2020). For comparison, susceptibility to gentamicin and ciprofloxacin is shown in Tables 1 and 2. Liofilchem® MIC Test Strips were put on inoculated MH agar plates and incubated at 37 °C for 18 h, quality control *S. aureus* ATCC 29,213.

**Table 1:** Minimum inhibitory/bactericidal concentration (MIC/MBC, mg/ml mean ? SD) of plant compouds against canine skin staphylococci

Plant compounds	Strain	Staphylococcus							
		aureus		haemolyti-cus	pseudintermedius		capitis	epidermidis	hominis
		CE24	BO4	Ri3	TB13	OG9	ME17	AL7	AG3
Betulin	MIC	16.0 ± 0.0	16.0 ± 0.0	16.0 ± 0.0	16.0 ± 0.0	16.0 ± 0.0	8.0 ± 0.0	16.0 ± 0.0	8.0 ± 0.0
	MBC	-	-	-	-	-	-	-	-
Curcumin	MIC	4.0 ± 0.0	2.0 ± 0.0	2.7 ± 1.2	2.0 ± 0.0	2.0 ± 0.0	1.7 ± 0.6	2.0 ± 0.0	1.8 ± 1.9
	MBC	10.7 ± 4.6	10.7 ± 4.6	8.0 ± 0.0	-	13.3 ± 4.6	8.0 ± 0.0	16.0 ± 0.0	8.0 ± 0.0
Glycyrrhizic acid	MIC	6.7 ± 2.3	8.0 ± 0.0	8.0 ± 0.0	8.0 ± 0.0	8.0 ± 0.0	16.0 ± 0.0	8.0 ± 0.0	6.7 ± 2.3
	MBC	-	-	-	16.0 ± 0.0	16.0 ± 0.0	16.0 ± 0.0	-	16.0 ± 0.0
Guaiazulene	MIC	16.0 ± 0.0	16.0 ± 0.0	-	-	-	16.0 ± 0.0	-	16.0 ± 0.0
	MBC	16.0 ± 0.0	-	-	-	-	-	-	-
Piperine	MIC	16.0 ± 0.0	-	-	-	-	-	-	16.0 ± 0.0
	MBC	-	-	-	-	-	-	-	-
Quercetin	MIC	-	-	16.0 ± 0.0	16.0 ± 0.0	-	10.7 ± 4.6	16.0 ± 0.0	8.0 ± 0.0
	MBC	-	-	-	-	-	16.0 ± 0.0	-	-
Quinine	MIC	0.33 ± 0.14	0.33 ± 0.14	0.83 ± 0.29	0.67 ± 0.29	1.00 ± 0.00	0.58 ± 0.38	1.67 ± 0.58	1.33 ± 0.57
	MBC	0.58 ± 0.38	1.00 ± 0.87	2.00 ± 1.73	2.00 ± 1.73	1.33 ± 0.57	0.67 ± 0.29	2.33 ± 1.53	1.67 ± 0.58
Tannic acid	MIC	0.25 ± 0.00	0.33 ± 0.14	1.00 ± 0.00	0.12 ± 0.00	0.12 ± 0.00	0.33 ± 0.14	0.25 ± 0.00	0.04 ± 0.02
	MBC	1.33 ± 0.57	1.33 ± 0.57	1.00 ± 0.00	1.67 ± 0.58	0.67 ± 0.29	0.33 ± 0.14	0.83 ± 0.29	0.25 ± 0.00
Ciprofloxacin^**a**^	μg/ml	0.19	0.125	0.125	0.125	0.5	0.25	0.125	0.25
Gentamicin		0.75	0.5	0.125	0.19	0.25	0.19	0.19	0.125

^a^tested by MIC Test Strips (Liofilchem); “-” no antimicrobial activity detected up to tested range 16 mg/ml

**Table 2:** Minimum inhibitory/bactericidal concentration (MIC/MBC, mg/ml mean ? SD) of plant compouds against other skin bacteria

Plant compounds	Strain	Streptococcus canis	Enterococcus faecium	Bacillus cereus	Pseudomonas aeruginosa	Klebsiella oxytoca	Escherichia coli
		BS10	AA14	AE13	BAS1	AX10	EL13
Betulin	MIC	16.0 ± 0.0	16.0 ± 0.0	6.7 ± 2.3	6.7 ± 2.3	8.0 ± 0.0	10.7 ± 4.6
	MBC	-	-	-	-	-	-
Curcumin	MIC	2.0 ± 0.0	3.3 ± 1.2	1.7 ± 0.6	6.7 ± 2.3	16.0 ± 0.0	16.0 ± 0.0
	MBC	-	16.0 ± 0.0	-	-	-	-
Glycyrrhizic acid	MIC	16.0 ± 0.0	16.0 ± 0.0	1.7 ± 0.6	8.0 ± 0.0	16.0 ± 0.0	16.0 ± 0.0
	MBC	-	-	-	-	-	-
Guaiazulene	MIC	-	16.0 ± 0.0	10.7 ± 4.6	16.0 ± 0.0	13.3 ± 4.6	16.0 ± 0.0
	MBC	-	-	-	-	-	-
Piperine	MIC	-	-	1.2 ± 0.8	16.0 ± 0.0	4.3 ± 3.1	16.0 ± 0.0
	MBC	-	-	-	-	-	-
Quercetin	MIC	16.0 ± 0.0	16.0 ± 0.0	8.0 ± 0.0	10.7 ± 4.6	16.0 ± 0.0	16.0 ± 0.0
	MBC	-	-	-	-	-	-
Quinine	MIC	1.0 ± 0.0	16.0 ± 0.0	5.3 ± 2.3	16.0 ± 0.0	2.7 ± 1.2	3.3 ± 1.2
	MBC	4.0 ± 0.00	-	16.0 ± 0.0	16.0 ± 0.0	4.7 ± 2.5	5.3 ± 1.9
Tannic acid	MIC	1.0 ± 0.0	16.0 ± 0.0	0.12 ± 0.00	1.0 ± 0.0	8.0 ± 0.0	1.0 ± 0.0
	MBC	2.0 ± 0.0	-	4.0 ± 0.0	-	-	8.0 ± 0.0
Ciprofloxacin^**a**^	μg/ml	NT	4	NT	4	0.03	0.03
Gentamicin		0.5	8	0.5	0.5	0.25	0.25

^a^tested by MIC Test Strips (Liofilchem); NT - not tested

After this study, the most potent compounds in terms of antibacterial activity (tannic acid and quinine) were tested in combination in ratios of 1:1, 1:2 and 2:1, starting with 32:32 mg/ml continuing with 16:32 and 32:16 mg/ml. The MIC and MBC were determined in triplicates as described above. Five canine skin isolates representing different genera (*B. cereus* AE13, *K. oxytoca* AX10, *P. aeruginosa* BAS1, *S. aureus* CE24, *S. pseudintermedius* TB13) and three collection strains (*E. coli* CCM 3954, *S. aureus* CCM 4223 and *S. pseudintermedius* CCM 4710) were used for testing purposes.

### Creams preparation

Tannic acid (403040-50G, Sigma-Aldrich, USA) was diluted in purified water, and quinine (22620-5G, Sigma-Aldrich) was diluted in a mixture of 96% ethanol (Centralchem, Slovakia) and 99% acetic acid (Centralchem). Both solutions were mixed and then gradually mixed into the cream base AquaNeoFarm® (Fagron, Czech Republic) – cream E or Ambiderman (Galvex, Slovakia) – cream F. Table 3 lists the concentrations of individual substances. The AquaNeoFarm® cremor is an emulsifying hydrophilic cream base (o/w) with a non-ionic emulsion system. The cream is composed of stearic acid 50, cetostearyl alcohol, glycerol monostearate 40–55, Slovasol 2430, paraffin oil, paraffin wax, methylparaben, propylparaben, 85% glycerol and purified water. The water of another hydrophilic liquid can be mixed directly into the base (in an amount corresponding to their solubility), whereas substances of a lipophilic nature must be heated (40–50 °C) together with the cream before mixing. Ambiderman is composed of cetostearyl alcohol, Slovasol 2430, paraffin oil, paraffin wax, Carbopol 940, methylparaben, propylparaben, trolamine, propylene glycol and purified water. It is a hydrophilic cream base. The determination of MIC and MBC for (a) cream E and F; (b) cream E and F without solvents (acetic acid, ethanol) but with active compounds tannic acid and quinine; and (c) cream E and F without active compounds (tannic acid, quinine) but with solvents (ethanol, acetic acid) was performed similarly by the broth microdilution method using resazurin (in triplicate) with the initial concentration 250 mg/ml of cream in MH broth (range 250–0.98 mg/ml). For this detection, we used the same bacterial strains as in the testing of tannic acid and quinine in combination. Positive control is growth control (broth with bacteria without cream), whereas negative control is sterility control (cream with broth).

**Table 3:** Composition of cream E and F (the weight in mg)

Excipients	Cream E	Cream F
quinine	200	200
tannic acid	100	100
acetic acid 99%	100	100
ethanol 96%	1800	1800
purified water	900	900
AquaNeoForm	6900	
Ambiderman		6900

### Organoleptic characteristics

The physical attributes of both formulations with ingredients and cream bases themselves were evaluated for their organoleptic characteristics, such as appearance, colour, texture, phase separation and homogeneity. These characteristics were assessed by visual inspection (three evaluators). Homogeneity and texture were determined by pressing a small amount of cream between the thumb and index finger. The texture and homogeneity of the formulations were evaluated based on their consistency and the presence of coarse particles. Additionally, immediate skin feeling characteristics, such as stiffness, grittiness and greasiness, were also assessed.

### Spreadability

To determine the spreadability of the formulations, the spreading diameter of 1 g of sample was measured between two horizontal glass plates (10 cm x 10 cm, Chen et al. 2016). The weight of the upper glass plate was 50 g. Three measurements were carried out: without weight and with a standard weight of 50 and 100 g applied to the upper plate after one minute each. Each formulation underwent three tests.

### pH measurement

The pH values of the two formulations with ingredients and cream bases were determined by dispersing 1 g of each in 25 ml of purified water. This was done using a Seven Compact pH/Ion S220 pH metre (Mettler-Toledo AG, USA), and the measurements were taken in triplicate. Prior to each use, the pH meter was calibrated using standard buffer solutions (pH 4, 7 and 10).

### Detection of microbial inactivation time

The inactivation time was tested in duplicate by incubation of a mixture of cream E and F and tested bacteria using the method described by Han et al. (2015) with minor modification. The bacterial suspension in MH broth was adjusted to 10^4^ CFU/ml. Two g of cream was dissolved in 6 ml of the prepared bacterial suspension and the mixture was incubated for 1, 2, 3 and 6 h at 37 °C. After incubation, serial ten-fold dilution was prepared, and 100 µl of appropriate dilution was inoculated on Brain Heart Infusion agar plates (Becton and Dickinson, USA). The plates were incubated for 24 h at 37 °C, and the cultured colonies were then counted. As positive controls, a bacterial suspension (6 ml) with MH broth (2 ml) was incubated and inoculated using the same procedures.

### Statistical analysis

All data were expressed as mean ± standard deviation. Analysis of the antimicrobial effect was performed using one way ANOVA followed by the Tukey test using GraphPad Prosim5 software. Synergy test was performed to determine the impact on potency of the combination of compounds (tannic acid and quinine in tested ratios) in comparison to their individual activities using results for bacterial strains tested in both MIC assays (*K. oxytoca* AX10, *S. aureus* CE24, *P. aeruginosa* BAS1). A Fractional Inhibitory Concentration (FIC) index value lower than 0.5 was evaluated as synergy.

## Results

### Minimum inhibitory/bactericidal concentrations of plant compounds

Tables 1 and 2 show the results of MIC and MBC determination for the eight different plant compounds. In general, their inhibitory activity was observed in the concentration range from 0.04 to more than 16 mg/ml or no activity (MIC) and from 0.25 to more than 16 mg/ml or no activity (MBC), respectively. Based on the MIC and MBC averages for all tested strains according to the type of plant compound, the most potent seems to be tannic acid (MIC average 2.1 mg/ml), followed by quinine (3.7 mg/ml) and curcumin (4.6 mg/ml). According to ANOVA statistical analysis, the MIC values of quinine were significantly lower (*P* < 0.05) compared to all the other tested compounds (except tannic acid) and in all tested strains except *E. faecium* AA14 and *B. cereus* AE13, where curcumin showed a lower MIC. Tannic acid has similarly significantly lower MIC values (*P* < 0.05) compared to other compounds (except quinine) and in all strains except *E. faecium* AA14 and *B. cereus* AE13. In contrast, many plant compounds were active against the tested bacteria only in concentrations of 16 mg/ml or have no activity at tested concentrations (piperine, quercetin, guaiazulene). Most of the tested compounds seem to be more active against Gram-positive bacteria (tannic acid, quinine, curcumin, glycyrrhizic acid), but some were more active (lower MIC) against Gram-negative bacteria (betulin, piperine). The strain most susceptible to plant compounds in general was *B. cereus* AE13, while *E. faecium* AA14 was the most resistant strain (the highest MICs) among the tested bacteria.

### Antibacterial effect of mixture of plant compounds

The two most potent plant compounds were selected to test their inhibitory effect against eight bacterial strains in combination (Table 4). The results for tannic acid and quinine in three different ratios (1:1, 1:2, 2:1) showed relatively strong antimicrobial effect, with MIC ranging between < 0.06:0.06 (1:1) and 32.0:16.0 (2:1) mg/ml and MBC ranging from < 0.06:0.03 (2:1) and > 32:16 (2:1) mg/ml. More susceptible among the tested bacteria were *B. cereus* and *S. pseudintermedius*; however, the sensitivity or resistance of the tested bacteria was different at different ratios. When comparing the antibacterial effect according to the ratios used, the lowest MIC and MBC values and thus the highest effectivity was observed for the ratio 1:2 tannic acid and quinine (the lowest fractional inhibitory concentration index FIC ranging from 0.95 to 11.8; however, synergy was not noted), followed by a 1:1 ratio. In contrast, the highest MIC and MBC was detected at a ratio of 2:1 for all tested bacteria except for *S. pseudintermedius* CCM 4710. The lowest fractional inhibitory concentration index (FIC) was observed for 1:2 ratio and ranged from 0.95 (*K. oxytoca* AX10, by 1:2 ratio) to 11.8 (*S. pseudintermedius* CE24, 1:2).

**Table 4:** Minimum inhibitory/bactericidal concentration of tannic acid and quinine (in mg/ml) tested in different ratio (1:1, 1:2, 2:1; mean ± SD)

Tested bacteria	1:1		1:2		2:1	
	MIC	MBC	MIC	MBC	MIC	MBC
*Bacillus cereus* AE13	< 0.06:0.06	< 0.06:0.06	0.7:1.3 ± 0.2:0.5	1.0:2.0 ± 0.0:0.0	2.0:1.0 ± 0.0:0.0	24.0:12.0 ± 11.3:5.7
*Escherichia coli* CCM 3954	7.3:7.3 ± 1.5:1.5	10.7:10.7 ± 3.8:3.8	1.3:2.7 ± 0.5:0.9	2.0:4.0 ± 1.0:2.0	16.0:8.0 ± 0.0:0.0	26.7:13.3 ± 7.5:3.8
*Klebsiella oxytoca* AX10	5.0:5.0 ± 2.2:2.2	6.0:6.0 ± 2.0:2.0	1.1:2.2 ± 0.4:0.9	2.3:4.7 ± 1.2:2.5	32.0:16.0 ± 0.0:0.0	>32.0:16.0
*Pseudomonas aeruginosa* BAS1	4.3:4.3 ± 3.0:3.0	13.3:13.3 ± 3.8:3.8	0.9:1.8 ± 0.2:0.4	5.3:10.7 ± 1.9:3.8	16.0:8.0 ± 0.0:0.0	18.7:9.3 ± 6.0:3.0
*Staphylococcus aureus* CCM 4223	7.3:7.3 ± 1.5:1,5	8.0:8.0 ± 0.0:0.0	1.8:3.6 ± 0.4:0.7	3.3:6.7 ± 0.9:1.9	12.0:6.0 ± 4.0:2.0	29.3:14.7 ± 6.0:3.0
*Staphylococcus aureus* CE24	3.0:3.0 ± 1.0:1.0	6.0:6.0 ± 2.0:2.0	1.2:2.3 ± 0.4:0.7	1.8:3.7 ± 1.1:2.1	8.0:4.0 ± 0.0:0.0	12.0:6.0 ± 8.9:4.5
*Staphylococcus pseudintermedius* CCM 4710	1.8:1.8 ± 0.4:0.4	3.7:3.7 ± 0.7:0.7	0.8:1.6 ± 0.2:0.5	2.7:5.3 ± 1.4:2.7	< 0.06:0.03	2.2:1.1 ± 1.8:0.9
*Staphylococcus pseudintermedius* TB13	3.3:3.3 ± 0.9:0.9	4.7:4.7 ± 1.5:1.5	0.4:0.8 ± 0.3:0.7	1.3:2.5 ± 0.8:1.5	10.7:5.3 ± 5.5:2.7	24.0:12.0 ± 8.0:4.0

### Antibacterial effect and organoleptic characteristics of cream formulation

The 1:2 ratio of tannic acid and quinine showing the strongest antibacterial activity was used for preparation of a cream. Cream formulations were completed with the addition of 1% (w/w) pure acetic acid, which showed the best antibacterial effect in our previous study (Štempelová et al. 2023). The creams E and F differed only in the cream base used (in cream E AquaNeoFarm and in cream F Ambiderman).

**Fig. 1:**
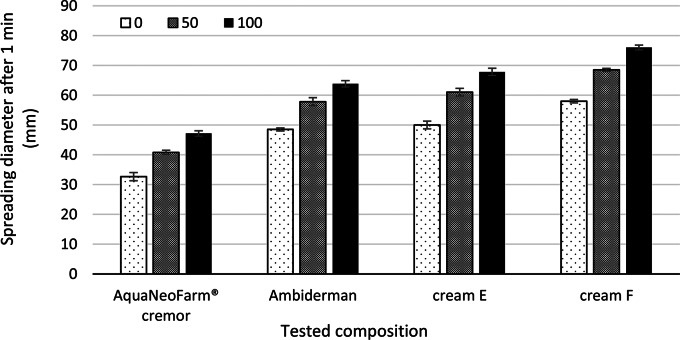
Spreading diameter of cream formulations (mm) after 1 min with a standard weight of 50 g and 100 g

Table 5 presents the organoleptic properties of the topical formulations, which include their physical appearance, colour, texture, phase separation, homogeneity and immediate skin feels. The results indicated that the creams containing active ingredients were comparable to the cream bases in terms of appearance and texture. Additionally, all formulations were homogeneous and did not show any signs of phase separation. The cream bases had a white colour, whereas the formulations had a beige colour with a pink tint. The cream bases were greasier and slightly stiff, whereas the formulations were very light and smooth and did not leave a greasy film on the skin. The even distribution of a cream on the skin, or spreadability, is a crucial factor in ensuring that a standard dose of a medicated formulation is administered and in determining the efficacy of a topical therapy. Figure 1 displays the formulations’ observed spreading values, or diameters, after one minute pressure of only an upper glass plate (0 g), 50 and 100 g standard weights. These values represent the ease with which the formulations can spread on the application surface with minimal force. The results revealed that creams with active ingredients had a higher spreadability than the original cream base products, as a relatively large amount of liquid had been incorporated into them.

**Table 5:** Physicochemical evaluation of selected topical formulations

Formulation	Physical appearence	Color	Texture	Phase separation	Homogeneity	Immediate skin feels
E	opaque	beige with a pink tint	smooth	no	homogeneous	very light and smooth, no remaining film
F	opaque	beige with a pink tint	smooth	no	homogeneous	very light and smooth, no remaining film
AquaNeoFarm® cremor	opaque	white	smooth	no	homogeneous	stiffer, more greasing, remains greasy film
Ambiderman	opaque	white	smooth	no	homogeneous	light, slightly greasing

As expected, the addition of active ingredients caused a decrease in the pH of the formulations. The average pH value of cream F was 4.21 ± 0.00 and of cream E 4.35 ± 0.03. The pH value of the cream bases was as follows: Ambiderman 6.98 ± 0.00, AquaNeoFarm® 4.95 ± 0.01.

The MIC for cream F ranged between < 0.98 and 62.5 mg/ml, whereas the MIC for cream E ranged between < 0.98 and 83.3 mg/ml (Table 6). Cream F showed significantly lower MIC (*P* < 0.01) for several tested bacteria (*P. aeruginosa* BAS1, *S. aureus* CCM 4223, *S. aureus* CE24) compared to cream E. The MBC was considerably high for *B. cereus*; however, the MIC was under 0.98 mg/ml, which means bacteriostatic effect against this bacterium for both creams. When we compare creams with full composition and creams without solvents (Table 6), a lower MIC for some bacterial strains (cream F vs. cream F without solvents – *S. aureus* CCM 4223, *S. aureus* CE24, *P* < 0.001) was observed for creams with full composition. This means that ethanol and acetic acid are necessary to solve these two plant compounds. In addition, MIC testing of the cream bases with solvents showed no antimicrobial effect against the tested bacteria up to 125 mg/ml, thus confirming no activity of the solvents in the cream formulations.

**Table 6:** Minimum inhibitory/bactericidal concentration (in mg/ml) of cream E and F with or without acetic acid and ethanol (-AA, E) against selected bacteria (mean ± SD)

Tested bacteria	MIC		MBC		MIC		MBC	
	Cream E	Cream F	Cream E	Cream F	E-AA,E	F-AA,E	E-AA,E	F-AA,E
*Bacillus cereus* AE13	< 0.98	< 0.98	-	-	3.3 ± 1.1	3.9 ± 0.0	-	-
*Escherichia coli* CCM 3954	62.5 ± 0.0	62.5 ± 0.0	104.0 ± 29.0	62.5 ± 0.0	125.0 ± 0.0	62.5 ± 0.0	145.8 ± 95.5	-
*Klebsiella oxytoca* AX10	72.9 ± 47.7	41.7 ± 18.0	125.0 ± 0.0	62.5 ± 0.0	125.0 ± 0.0	125.0 ± 0.0	145.8 ± 95.5	-
*Pseudomonas aeruginosa* BAS1	83.3 ± 36.1	7.8 ± 0.0	125.0 ± 0.0	31.3 ± 0.0	125.0 ± 0.0	26.0 ± 9.0	166.7 ± 72.2	-
*Staphylococcus aureus* CCM 4223	62.5 ± 0.0	13.0 ± 4.5	125.0 ± 0.0	15.6 ± 0.0	62.5 ± 0.0	62.5 ± 0.0	166.7 ± 72.2	-
*S. aureus* CE24	3.3 ± 1.1	7.8 ± 0.0	20.8 ± 9.0	10.4 ± 4.5	125.0 ± 0.0	62.5 ± 0.0	166.7 ± 72.2	-
*S. pseudintermedius* CCM 4710	< 0.98	< 0.98	0.98 ± 0.0	3.3 ± 0.9	7.8 ± 0.0	3.9 ± 0.0	26.0 ± 9.0	36.4 ± 23.9
*S. pseudintermedius* TB13	< 0.98	< 0.98	0.98 ± 0.0	0.98 ± 0.0	1.9 ± 1.7	62.5 ± 0.0	26.0 ± 9.0	20.8 ± 9.0

“-” no antimicrobial activity detected up to tested range 250 mg/ml

### Detection of microbial inactivation time

The results for microbial inactivation time analysis are shown in Table 7. The bacterial strains used were inactivated between 1 and 6 h of viable cells determination. The most susceptible bacteria to tested cream formulation F was *P. aeruginosa* BAS1, which was already inhibited after only 1 h of incubation (< 10^1^ CFU/ml). After 3 h of mixture incubation, all tested bacteria were in undetectable counts except for the skin isolate *S. aureus* CE24, still with viable cells of 10^2^ CFU/ml. At the last sampling (6 h), all tested bacteria were inhibited, while visible growth was observed for the control samples (10^7–10^ CFU/ml).

**Table 7:** Detection of microbial inactivation time of cream F formulation for selected bacteria (in log CFU/ml, mean ± SD)

Tested bacteria	Sampling (h)									
	0 h		1 h		2 h		3 h		6 h	
	Ex	C	Ex	C	Ex	C	Ex	C	Ex	C
*Bacillus cereus* AE13	4.5 ± 0.1	4.3 ± 0.6	2.5 ± 0.1	4.2 ± 0.2	< 1.0	4.4 ± 0.4	< 1.0	5.5 ± 0.1	< 1.0	7.9 ± 0.4
*Escherichia coli* CCM 3954	4.4 ± 0.2	4.3 ± 0.1	3.7 ± 0.1	4.3 ± 0.1	2.4 ± 0.2	5.4 ± 0.1	< 1.0	6.5 ± 0.2	< 1.0	8.2 ± 0.1
*Klebsiella oxytoca* AX10	4.7 ± 0.0	4.4 ± 0.1	3.3 ± 0.1	4.6 ± 0.1	< 1.0	5.1 ± 0.1	< 1.0	6.2 ± 0.2	< 1.0	9.6 ± 0.3
*Pseudomonas aeruginosa* BAS1	4.9 ± 0.1	4.7 ± 0.0	< 1.0	4.4 ± 0.1	< 1.0	5.0 ± 0.1	< 1.0	6.2 ± 0.1	< 1.0	9.9 ± 0.3
*Staphylococcus aureus* CCM 4223	4.4 ± 0.1	4.4 ± 0.1	3.9 ± 0.0	4.4 ± 0.1	1.8 ± 0.0	4.4 ± 0.1	< 1.0	5.3 ± 0.0	< 1.0	9.9 ± 0.4
*Staphylococcus aureus* CE24	4.3 ± 0.1	4.1 ± 0.2	4.0 ± 0.1	4.3 ± 0.1	3.3 ± 0.1	4.6 ± 0.1	2.4 ± 0.1	4.8 ± 0.1	< 1.0	9.4 ± 0.4
*Staphylococcus pseudintermedius* CCM 4710	4.9 ± 0.0	4.8 ± 0.1	2.7 ± 0.1	4.6 ± 0.0	< 1.0	5.0 ± 0.2	< 1.0	5.6 ± 0.2	< 1.0	8.5 ± 0.4
*Staphylococcus pseudintermedius* TB13	4.6 ± 0.1	4.6 ± 0.1	4.4 ± 0.1	4.4 ± 0.0	2.8 ± 0.1	4.6 ± 0.1	< 1.0	4.8 ± 0.2	< 1.0	9.2 ± 0.1

Ex-experimental, C-control

## Discussion

In our study, we included less expensive compounds with many therapeutic benefits. The most inhibitory active (the lowest MIC) was tannic acid, which is a naturally occurring plant polyphenol that can be found in practically all aerial plant tissues (especially in tea leaves, gallnuts and acacia bark). It has been reported that tannic acid has natural antioxidant, antimicrobial and antiviral activity (von Martius et al. 2012). The study of Dong et al. (2018) also showed that tannic acid inhibited *S. aureus* biofilm formation effectively and could therefore be used as potential candidate for the eradication of methicillin-resistant *S. aureus*. Their results revealed that tannic acid might directly bind to peptidoglycan in the cell wall and interfere with its integrity. Quinine, the second most effective compound in our study, is a medication that has been used to treat malaria caused by *Plasmodium falciparum* for centuries. It is an alkaloid isolated from the bark of the cinchona tree and possesses anti-inflammatory, antioxidant and anticancer properties in addition to its antimalarial activity. According to our MIC results for quinine, higher values were observed for Gram-negative than for Gram-positive bacteria. Antika et al. (2020) reported similar MIC concentrations for quinine against human pathogenic bacteria (*E. coli*, *P. aeruginosa*, *S. aureus*), ranging between 0.25 and 0.5%. Their study also showed that quinine derivates were not as effective as quinine itself. Another compound showing high efficiency, especially against Gram-positive bacteria, is curcumin. Curcumin is a bright yellow bioactive chemical produced by the plant species *Curcuma longa* (turmeric), a member of the ginger family Zingiberaceae. It is a diarylheptanoid belonging to the group curcuminoids, which are phenolic pigments responsible for yellow colour of turmeric. Its only disadvantage is that it is of low aqueous solubility and poor oral bioavailability, a problem currently being addressed by efforts to increase it through the preparation of nanoparticles (Rajsekhar et al. 2015). Curcumin is commonly used as a spice, as a dietary supplement, as an ingredient in cosmetics or as a flavouring or colouring agent for foods (food additive E100 in EU). Curcumin’s antimicrobial activity involves inhibition of cell proliferation through disrupting the guanosine triphosphatase (GTPase) activity of FtsZ protofilaments, which play critical role in bacterial cytokinesis (Rai et al. 2008), although another study has shown that curcumin inhibits bacterial surface protein sortase A and prevents cell adhesion to fibronectin (Park et al. 2005).

The difference in the efficacy of the antibacterial effect against Gram-positive and Gram-negative bacteria is often ascribed to the presence of an outer membrane in Gram-negative bacteria, which is an efficient barrier for compound entry. However, some of our studied plant-derived compounds, such as betulin or piperine, showed better activity against Gram-negative strains. Therefore, an analysis needs to be done of the relationship between the chemical structure and its biological properties.

The two most inhibitory active compounds (tannic acid and quinine) were combined to achieve possible synergistic activity. Three different ratios (1:1, 1:2, 2:1) against eight selected bacteria were tested. The results indicated that MIC and MBC were lower for some bacteria (*B. cereus*, *K. oxytoca*, *P. aeruginosa*), especially in a 1:2 (tannic acid:quinine) combination than the MBC/MIC for these compounds alone. In this ratio (1:2), all tested bacteria were inhibited at the concentration 5.3:10.7 mg/ml (MBC), which is a lower concentration than for most of the tested plant compounds. In testing the 1:2 ratio, no notable differences in the MIC and MBC concentrations against Gram-positive and Gram-negative bacteria were seen. Similarly, Antika et al. (2020) also reported no visible difference in inhibitory concentrations of quinine against human pathogenic Gram-negative (*E. coli*, *P. aeruginosa*) and Gram-positive (*B. cereus*, *S. aureus*) bacteria. On the other hand, the antibacterial study of tannins obtained by extraction from *Anthemis praecox* Link against six different bacterial species showed that Gram-positive species were more susceptible than Gram-negative species to these extracts (Belhaoues et al. 2020). It is necessary to say that many of such studies used crude plant extracts containing other phenolic compounds aside from tannic acid.

Although the synergistic effect of our tested combinations was not achieved, some researchers have observed a synergistic effect in the combination of a plant compound (e.g. tannic acid) with antibiotics (Tintino et al. 2016). These authors concluded that tannic acid may work as an inhibitor of the NorA efflux pump, considered to be the main mechanism responsible for its antibacterial activity. Based on the antibacterial activity results showing the 1:2 ratio of tannic acid and quinine as having the highest antimicrobial efficacy, both compounds were incorporated in such a ratio in two cream formulations. It has long been known that mixing quinine and tannic acid produces a precipitate, and these ingredients are considered incompatible in pharmacy (Chalabala et al. 1961). The main problem is tannic acid, which in addition to quinine also precipitates with salts of heavy metals, proteins, gelatin, gum arabic, starch, zinc oxide and with many alkaloids to form insoluble tannates. To increase the solubility of both components, we reduced the pH using acetic acid and increased the proportion of alcohol in the solution. The solution was incorporated into two cream bases AquaNeoFarm® cremor (Fagron, Czech Republic) – cream E and Ambiderman (Galvex, Slovakia) – cream F. Despite comparable MIC and MBC results for both creams, cream F showed lower MIC for three and lower MBC for five tested bacteria compared to cream E. This difference could be attributed to better solubility of the compounds in the Ambiderman cream base. The highest MIC of cream F was obtained for *E. coli* (62.5 mg/ml). Han et al. (2015) evaluating the in vitro efficacy of a topical skin cream containing a mixture of emu oil, jojoba oil, avocado oil and tea tree oil against canine skin pathogens reported MIC, MBC only for *S. pseudintermedius* (MIC 0.23%, MBC 7.5%) and *Malassezia pachydermatis* (MIC 0.63%, MBC 5.0%). In our study, the MIC and MBC for two *S. pseudintermedius* strains were several times lower compared to these authors’ results. The advantage of cream formulation F could be not only the good antibacterial effect against Gram-positive and Gram-negative bacteria in a relatively short time (microbial inactivation time was 1–6 h) but also many other medical effects of the used compounds. The stimulation of epidermal regeneration, pain reduction, faster wound healing and astringent, antioxidant and antimutagenic properties were attributed to tannic acid (Hupkens et al. 1995; El-Sayed et al. 2014). The use of topical tannic acid and quinine in the proposed amount is inexpensive, easily available, entails no allergic reaction and has no systemic effects (El-Sayed et al. 2014). A recent study with topical application of quinine even showed its therapeutic effect in a mouse model of atopic dermatitis (Zhang et al. 2021), It significantly reduced symptoms like dryness, erythema, oedema and excoriation and thus repaired the skin barrier.

In summary, the current screening study shows the antibacterial effect of several plant-derived compounds, among which tannic acid and quinine were the most inhibitory compounds. The mixture of tannic acid and quinine showed the best antibacterial effect in the ratio of 1:2. A cream formulation with these compounds seems to be promising for further studies monitoring the cytotoxicity and overall therapeutic effect in infected skin lesions in dogs. In the case of its intended use as drug for otitis externa, it will be necessary to monitor their antimicrobial effect against yeasts as well. Since this study deals only with antimicrobial activity of these compounds, it would also be interesting to test their anti-inflammatory, antioxidant or other wound healing properties by using the proposed concentrations. Although the cream was effective in higher concentrations than the antibiotic, it does not contribute to the spread of antibiotic-resistant isolates. Moreover, the components costs are not as high.

## Data Availability

All data generated or analysed during this study are included in this published article.
